# CHALLENGES AND OPPORTUNITIES BRINGING DIGITAL INNOVATION TO ADULT DAY CENTERS: FEASIBILITY TESTING WITH CAREMOBI

**DOI:** 10.1093/geroni/igae098.1844

**Published:** 2024-12-31

**Authors:** Tina Sadarangani

**Affiliations:** New York University, New York, New York, United States

## Abstract

CareMobi is an mhealth application that allows adult day services centers to exchange information with their clients’ family caregivers and health care providers. CareMobi enables adult day center staff, who possesses in-depth knowledge of clients based on their day-to-day observations, to be active members of the care team. The application was developed using a user-centered design thinking approach that prioritized the needs of end-users. Initial prototype testing, which combined qualitative interviews followed by individual completion of the Technology Acceptance Model questionnaire, showed that likelihood of adopting CareMobi was high among adult day center staff (n=31). CareMobi was also perceived as having significant clinical value. We now present the results of preliminary feasibility and acceptability testing with the app itself in a rural Tennessee adult day center (n=9). We also identify factors that support successful implementation of technology within ADCs, including comprehensive support and education, as well as challenges to widespread adoption.

